# Use of PIRADS 2.1 to predict capsular invasion in patients with radiologic T3a prostate cancer

**DOI:** 10.3389/fonc.2023.1256153

**Published:** 2023-12-21

**Authors:** Wan Song, Kwang Jin Ko, Jae Kyung Lee, Minyong Kang, Hyun Hwan Sung, Hwang Gyun Jeon, Byong Chang Jeong, Seong IL. Seo, Seong Soo Jeon, Jae Hoon Chung

**Affiliations:** Department of Urology, Samsung Medical Center, Sungkyunkwan University School of Medicine, Seoul, Republic of Korea

**Keywords:** prostate cancer, magnetic resonance imaging, nomograms, prostatectomy, prostate special antigen

## Abstract

**Objective:**

Using multi-parametric magnetic resonance imaging (mpMRI) to identify patients with clinical T3a (cT3a) who were overestimated on mpMRI with final pathological T2 (pT2). To suggest that the neurovascular bundle (NVB) can be preserved by evaluating the characteristics of patients according to their pathological grade among cT3a prostate cancer (PCa) patients using mpMRI.

**Materials and methods:**

Patients who underwent robot-assisted laparoscopic radical prostatectomy (RALP) were retrospectively analyzed and those patients with clinical T3aN0M0 were enrolled. These enrolled patients were divided into a localized cancer group with pT2 PCa and a locally advanced group with pT3a or higher. Factors affecting the diagnosis of localized PCa after RALP in patients with cT3a PCa were evaluated.

**Results:**

Among the preoperative parameters of patients with cT3a PCa, the prostate specific antigen density (PSAD) (OR: 3.76, 95% CI: 1.85–7.64, p<0.001), international society of urological pathology (ISUP) grade (p<0.05), and index lesion size (OR: 1.44, 95% CI: 1.85–7.64, p<0.001) were significantly associated with pathological locally advanced PCa. Optimal cut-off values for prediction of pT3a or higher were 0.36 ng/mL2 for PSAD (sensitivity: 55.7%, specificity: 70.8%), 1.77 cm for index lesion size (sensitivity: 54.3%, specificity: 66.0%), and 2.5 for ISUP grading (sensitivity: 67.6%, specificity: 53.2%). For prediction of pT3a or higher among patients with cT3a PCa, a nomogram was developed using ISUP grade, index lesion size, and PSAD on prostate biopsy (area under the curve: 0.71, 95% CI: 0.670–0.754, p<0.001). PSAD less than 0.36 index lesion size less than 1.77 cm, and biopsy ISUP grade 1–2 are highly likely to indicate that there is no actual extracapsular extension in cT3a PCa patients.

**Conclusions:**

PSAD, ISUP, and index lesion size showed significant associations with the classification of pathologic localized and locally advanced PCa in patients with cT3a PCa. A nomogram including these features can predict the diagnosis of locally advanced PCa in patients with cT3a PCa.

## Introduction

Prostate cancer (PCa) is a common malignancy worldwide (1), and locally advanced prostate cancer (PCa) is a very high-risk disease with a high rate of biochemical recurrence, metastasis, and death (2). Although the optimal treatment for locally advanced PCa is not established (3, 4), robot-assisted laparoscopic radical prostatectomy (RALP) for locally advanced PCa is reported as a primary treatment with promising oncological outcomes (5, 6).

Although RALP may result in complete oncological resection, some patients could expect functional outcomes such as continence and potency because the neurovascular bundle (NVB) is preserved (7); continence and erectile function are two major concerns affecting the quality of life following surgery for PCa (8). The degree and methods of NVB preservation have been described in a variety of ways, but the grading by Srivastava et al. is widely used (9). The grade of NVB preservation is generally determined pre-operatively according to the clinical stage and risk. In particular, if findings of extracapsular extension (ECE) are observed on preoperative multiparametric magnetic resonance imaging (mpMRI), either both NVBs are sacrificed or only the NVB of the contralateral aspect is preserved (10). However, NVB preservation is an essential prerequisite for recovery of continence and potency after RALP, and when the NBV is sacrificed, the post-surgical quality of life is inevitably reduced (11).

Previous studies evaluating pre-biopsy mpMRI have demonstrated the effectiveness of mpMRI for tumor localization (12, 13). However, only a few studies have been conducted on the predictive accuracy of mpMRI for ECE. For prediction of ECE, mpMRI was shown to have higher accuracy than digital rectal examination, prostate specific antigen (PSA), and biopsy Gleason score (14). However, the sensitivity, specificity, PPV, and NPV of mpMRI for prediction of T3a have not been fully assessed, and there are many reports of inconsistent results (15). Therefore, the Prostate Imaging Reporting And Data System (PIRADS) v2.1 suggested some risk factors for T3a prostate cancer, such as capsular abutment, capsular irregularity, spiculation or retraction, neurovascular bundle asymmetry or thickening, obliteration of the recto-prostatic angle, tumor-capsular contact >10 mm, bulge or loss of capsule, and measurable extracapsular disease (16). Moreover, a study comparing this grading system to a Likert scale showed equivalent diagnostic performance, but also that the Likert scale does not rely on any specific criteria and is very subjective (17).

Despite these guidelines, the false positive rate of cT3a prostate cancer on mpMRI is reported as being as high as 50% (18). In this study, we aimed to use mpMRI to identify those patients with cT3a who are suitable for NVB preservation by evaluating the characteristics of patients with final pathological T2 or false positive cT3a.

## Materials and methods

### Patients & design

Patients who underwent RALP at our hospital between March 2020 and February 2023 were retrospectively assessed. RALP was performed by eight urologists. Among them, those patients with clinical T3aN0M0 who underwent RALP were identified. The inclusion criteria were patients who underwent RALP for prostate cancer and who had ECE on mpMRI. The exclusion criteria were neoadjuvant treatment such as androgen deprivation therapy, no preoperative mpMRI, and N1 status according to pathologic reports. The enrolled patients were divided into a group with localized pT2 PCa and a group with locally advanced PCa of pT3a or higher. Factors affecting the diagnosis of localized PCa after RALP in patients with cT3a PCa were evaluated.

**Figure 1:**
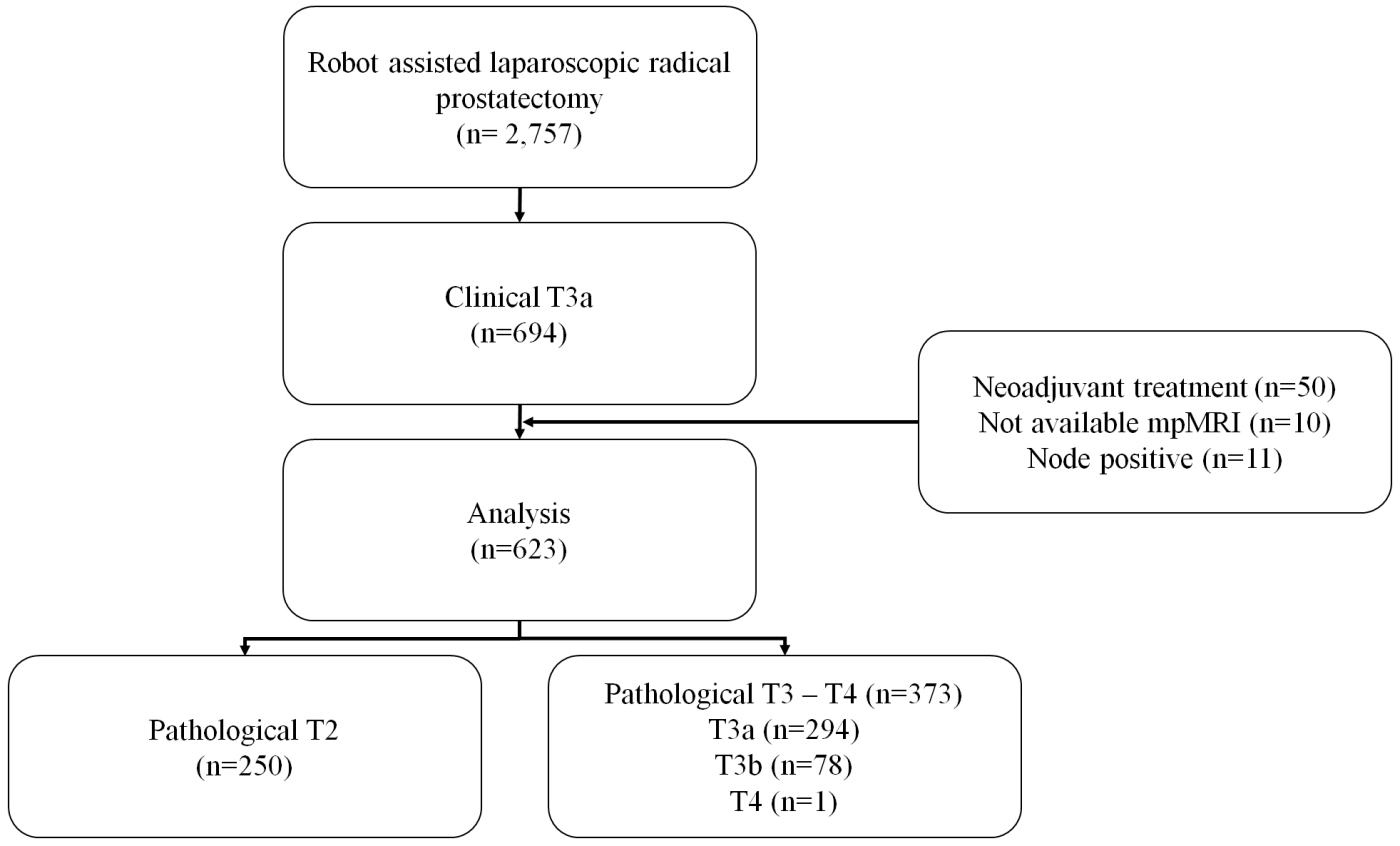
Flow diagram of patient inclusion.

Of the 2757 patients who underwent RALP during the study period, 694 patients with cT3a were identified. Fifty of the 694 patients underwent neoadjuvant ADT, and preoperative mpMRI data were not available for 10 further patients. In addition, 11 patients were excluded from this study because of pathologic N1 stage, leaving 623 patients for the final analysis. The final pathology results identified 40.13% (250/623) of these patients as having T2 prostate cancer. Of the remaining 373 patients, 294 were diagnosed with T3a, 78 with T3b, and one with T4 (**Figure 1**).

### Clinicopathological parameters

The baseline characteristics of the patients, including age at RALP, body mass index, underlying disease, familial history, 5α-reductase inhibitor (5ARI) administration history, PSA level, prostate volume (measured by MRI), prostate density, and biopsy results were assessed. On mpMRI, the PIRADS score, size of the index lesion, and number of PIRADS 3–5 lesions were recorded. Pathologic assessment was performed by two uro-pathologists. Pathologic reporting for biopsy and RALP specimens was randomly assessed by two uro-pathologists.

### mpMRI

Multiparametric MRI was performed using a 3.0-Tesla MRI scanner with a pelvic phased-array coil and without an endorectal coil. T2-weighted, diffusion-weighted, and dynamic contrast-enhanced sequences were acquired according to the minimum standards set by consensus guidelines (19). The mpMRI was analyzed by four uro-radiologists using PIRADS version 2.1 (16).

### Statistical analysis

The groups were compared using the chi-square test for categorical variables and the Student’s t-test for continuous variables. Logistic regression analysis was performed to evaluate preoperative factors affecting pT3a prostate cancer. The diagnostic ability of each parameter was assessed using the area under the curve (AUC) metric of the receiver operating characteristic (ROC). The cutoff value for the prediction of pT3a PCa was assessed by ROC curve analysis, and the cutoff value, sensitivity, specificity, positive predictive value (PPV), negative predictive value (NPV), and accuracy were estimated using Youden’s index method. Statistical analyses were performed using SPSS (version 21.0) and R 3.6.1 (Vienna, Austria; http://www.R-project.org). The pROC and rms packages in R were used to develop the nomogram. All two-sided p-values < 0.05 were considered statistically significant.

## Results

### Preoperative parameters

The mean PSA was 9.64 ± 7.51 ng/mL in the localized PCa group (pT2) and 15.42 ± 14.54 ng/mL in the locally advanced PCa group (pT3a to T4; p<0.001). The mean PSA density (PSAD) was 0.33 ± 0.24 ng/mL^2^ in the localized PCa group and 0.51 ± 0.41 ng/mL^2^ in the locally advanced PCa group (p<0.001). The international society of urological pathology (ISUP) grade for the Gleason score on prostate biopsy showed a significantly higher proportion of high grades in the locally advanced PCa group than in the localized PCa group (p<0.001). The mean proportion of positive cores among the total biopsy cores was 43.49% ± 21.35% in the localized PCa group and 53.29% ± 24.80% in the locally advanced group (p<0.001). On mpMRI, the localized group had a lower ratio of PIRADS 5 index lesions than the locally advanced group, and the mean index lesion size was 1.65 ± 0.56 cm in the localized group and 1.95 ± 0.93 cm in the locally advanced group (p<0.001) (**Table 1**).

**Table 1:** Baseline characteristics of clinical T3a prostate cancer (n=623).

Parameters		Localized (n=250)	Locally advanced (n=373)	p-value
Age, years		67.18 ± 7.22	67.93 ± 6.38	0.172
Body mass index, kg/m^2^		25.02 ± 2.98	25.05 ± 2.65	0.903
Hypertension, n (%)		127 (50.80)	170 (45.58)	0.201^†^
Diabetes mellitus, n (%)		60 (24.00)	78 (20.91)	0.363^†^
Dyslipidemia, n (%)		69 (27.60)	98 (26.27)	0.714^†^
Anti-coagulant, n (%)		70 (28.00)	111 (29.76)	0.636^†^
ASA, n (%)	1	20 (8.00)	19 (5.09)	0.431^†^
	2	191 (76.40)	294 (78.82)	
	3	39 (15.60)	59 (15.82)	
	4	0	1 (0.27)	
Smoking, n (%)	smoker	19 (7.60)	30 (8.04)	0.812^†^
	ex-smoker	154 (61.60)	237 (63.54)	
5-alpha reductase inhibitors		26 (10.40)	43 (11.53)	0.660^†^
Familial history, n (%)		23 (9.20)	25 (6.70)	0.252^†^
PSA, ng/mL		9.64 ± 7.51	15.42 ± 14.54	<0.001
Prostate volume, mL		32.28 ± 12.13	30.92 ± 13.80	0.196
PSA density		0.33 ± 0.24	0.51 ± 0.41	<0.001
ISUP grade	1	47 (18.80)	26 (6.97)	<0.001^†^
	2	86 (34.40)	95 (25.47)	
	3	59 (23.60)	104 (27.88)	
	4	46 (18.40)	115 (30.83)	
	5	12 (4.80)	33 (8.85)	
Positive biopsy cores, %		43.49 ± 21.35	53.29 ± 24.80	<0.001
Highest tumor percentage in core, %		60.76 ± 26.49	70.08 ± 25.36	<0.001
PIRADS of index lesion, n (%)	3	4 (1.60)	0	<0.001^†^
	4	82 (32.80)	75 (20.11)	
	5	164 (65.60)	298 (79.89)	
Size of index lesion, cm		1.65 ± 0.56	1.95 ± 0.93	<0.001
No. of PIRADS 3 to 5		1.33 ± 0.64	1.32 ± 0.61	0.841

ASA, American Society of Anesthesiologists; IIEF, international index of erectile function; PSA, prostate specific antigen; ISUP, International Society of Urological Pathology; PIRADS, Prostate Imaging Reporting And Data System.

Student t test, ^†^Chi-square test.

### Operative and pathologic outcomes

According to the final pathology reports, the rate of ISUP grades were significantly higher in the locally advanced PCa group (p<0.001). Compared to before RALP, the ISUP grade after surgery was downgraded in 21.35% (133/623), unchanged in 46.87% (292/623), and upgraded in 31.78% (198/623). Both perineural invasion and lymphovascular invasion showed significantly higher rates in the locally advanced group than in the localized PCa group (p<0.05). Tumor volume was also higher in the locally advanced group than in the localized group (15.31 ± 12.29% vs 28.05 ± 19.12%, p<0.001), but multifocality was lower in the locally advanced group (45.04%) than in the localized group (53.60%; p=0.036). The rate of margin involvement was 8.80% in the localized group and 37.00% in the locally advanced group (p<0.001). PSA persistence was present in 3.60% of the localized group and 14.75% of the locally advanced group (P<0.001), and biochemical recurrence occurred in 2.80% of the localized group and 11.53% of the locally advanced group (P<0.001) (**Table 2**).

**Table 2:** Surgical and oncological outcomes.

Parameters		Localized (n=250)	Locally advanced (n=373)	p-value
Operation time, minutes		151.48 ± 41.05	157.19 ± 41.08	0.090
Estimated blood loss, mL		142.44 ± 75.68	152.33 ± 86.58	0.132
NVB sparing, n (%)	Unilateral	121 (48.40)	209 (56.03)	0.171^†^
	Bilateral	46 (18.40)	57 (15.28)	
Pathologic Gleason score, ISUP grade	1	10 (4.00)	2 (0.54)	<0.001^†^
	2	123 (49.20)	97 (26.01)	
	3	80 (32.00)	141 (37.80)	
	4	22 (8.80)	62 (16.62)	
	5	15 (6.00)	71 (19.03)	
Perineural invasion, n (%)		233 (93.20)	365 (97.86)	0.004^†^
Lymphovascular invasion, n (%)		5 (2.00)	42 (11.26)	<0.001^†^
Multifocality, n (%)		134 (53.60)	168 (45.04)	0.036^†^
Tumor volume, %		15.31 ± 12.29	28.05 ± 19.12	<0.001
Margin involvement, n (%)		22 (8.80)	138 (37.00)	<0.001^†^
Biochemical recurrence, n (%)		7 (2.80)	43 (11.53)	<0.001^†^
PSA persistence		9 (3.60)	55 (14.75)	<0.001^†^
Nadir PSA		0.12 ± 0.93	0.44 ± 2.45	0.051

ISUP, International Society of Urological Pathology; NVB, neurovascular bundle; PSA, prostate specific antigen.

Student t test, ^†^Chi-square test.

### Logistic regression analysis

Among the preoperative parameters of the patients with cT3a PCa, PSAD (OR: 3.76, 95% CI: 1.85–7.64, p<0.001), ISUP grade (p<0.05), and index lesion size (OR: 1.44, 95% CI: 1.85–7.64, p<0.001) were significantly associated with pathologic locally advanced PCa (**Table 3**).

**Table 3:** Logistic regression analysis for locally advanced prostate cancer.

		Univariate analysis				Multivariate analysis			
		HR	95% CI		p-value	HR	95% CI		p-value
			Lower	Upper			Lower	Upper	
Age		1.02	0.99	1.04	0.172				
Body mass index		1.00	0.95	1.06	0.900				
Hypertension		0.81	0.59	1.12	0.201				
Diabetes mellitus		0.84	0.57	1.23	0.363				
Dyslipidemia		0.94	0.65	1.34	0.714				
Anti-coagulant		1.09	0.76	1.55	0.636				
ASA		1.20	0.85	1.69	0.292				
Smoking	smoker	1.15	0.60	2.19	0.677				
	ex-smoker	1.12	0.78	1.60	0.540				
5-alpha reductase inhibitors		1.12	0.67	1.88	0.660				
Familial history		0.71	0.39	1.28	0.254				
PSA density		6.87	3.58	13.21	<0.001	3.76	1.85	7.64	<0.001
ISUP grade	1	Reference							
	2	2.00	1.14	3.50	0.016	1.55	0.81	2.98	0.187
	3	3.19	1.79	5.67	<0.001	2.57	1.32	5.00	0.005
	4	4.52	2.51	8.14	<0.001	3.48	1.79	6.78	<0.001
	5	4.97	2.20	11.24	<0.001	3.54	1.39	9.02	0.008
Positive cores, %		1.02	1.01	1.03	<0.001	1.01	1.00	1.02	0.081
Highest tumor percentage in core		1.01	1.01	1.02	<0.001	1.00	1.00	1.01	0.504
PIRADS	3	Reference							
	4	1.48E+09			0.999				
	5	2.93E+09			0.999				
Size of index lesion		2.00	1.51	2.65	<0.001	1.44	1.06	1.96	0.020
No. of PIRADS 3 to 5		0.97	0.75	1.26	0.839				

ASA, American Society of Anesthesiologists; HR, hazard ratio; IIEF, international index of erectile function, PSA: prostate specific antigen; ISUP, International Society of Urological Pathology; PIRADS, Prostate Imaging Reporting And Data System.

### Prediction of locally advanced prostate cancer among cT3a patients

**Figure 2:**
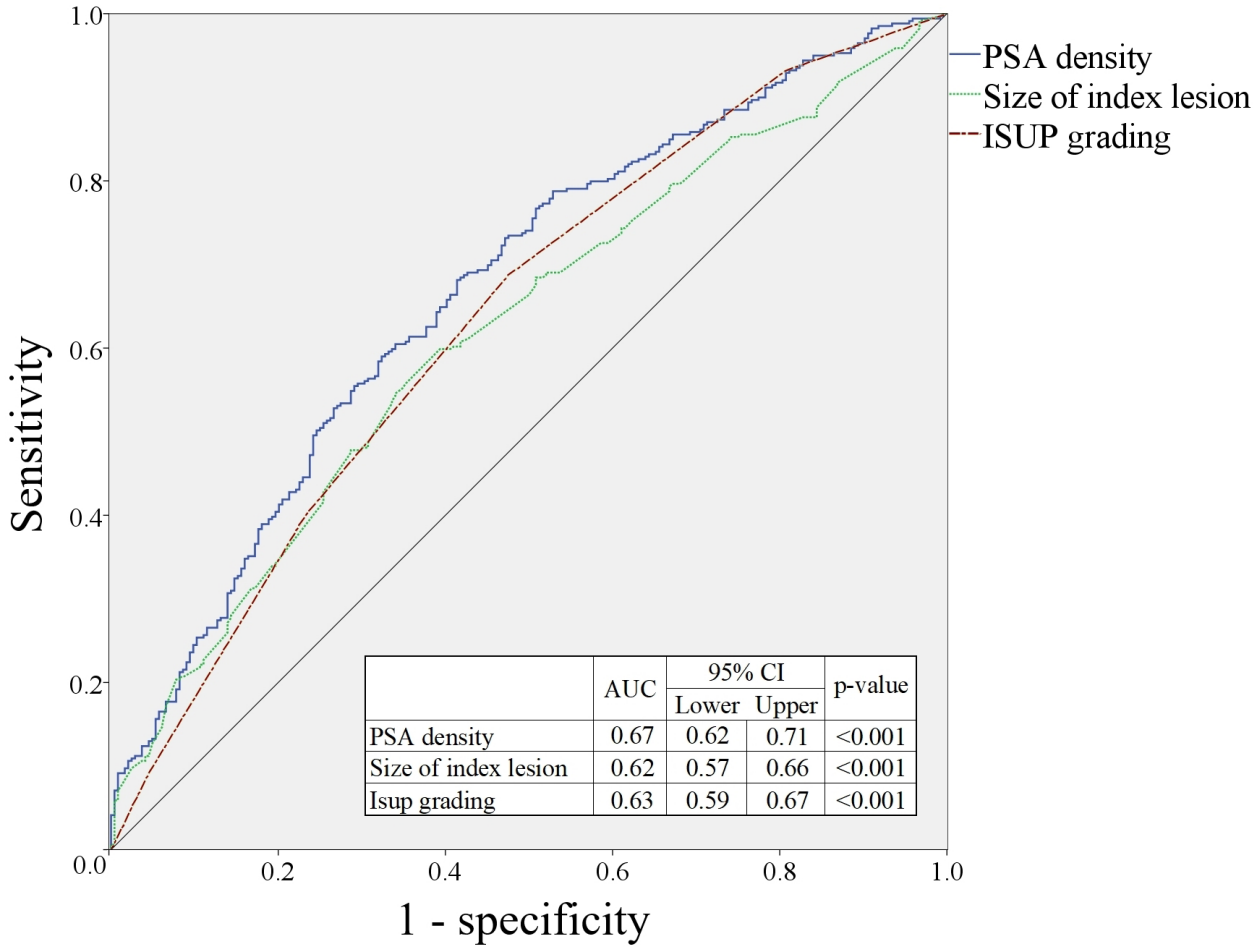
Prediction of locally advanced prostate cancer in patients with cT3a grade.

The areas under the curve (AUCs) for prediction of locally advanced prostate cancer were 0.67 (95% CI: 0.62–0.71, p<0.001) for PSAD, 0.62 (95% CI: 0.57–0.66, p<0.001) for index lesion size, and 0.63 (95% CI: 0.59–0.67, p<0.001) for ISUP grading (**Figure 2**). The optimal cut-off values were 0.36 (sensitivity: 55.7%, specificity: 70.8%) for PSAD, 1.78 (sensitivity: 54.3%, specificity: 66.0%) for index lesion size, and 2.5 (sensitivity: 67.6%, specificity: 53.2%) for ISUP grading (**Table 4**). PSAD less than 0.36, index lesion size less than 1.77 cm, and biopsy ISUP grade 1–2 are highly likely to indicate that there is no actual extracapsular extension in cT3a PCa patients.

**Table 4:** Cut-off values for prediction of pT3a prostate cancer.

Parameters	Cut-off	Sensitivity	Specificity	PPV	NPV	Accuracy	Youden index
PSA density	0.36	0.56	0.71	0.74	0.52	0.62	0.26
Size of index tumor	1.78	0.54	0.66	0.69	0.51	0.59	0.20
ISUP grade	2.5	0.68	0.53	0.76	0.43	0.62	0.21

PPV, positive predictive value; NPV, negative predictive value; PSA, prostate specific antigen; ISUP, International Society of Urological Pathology.

**Figure 3:**
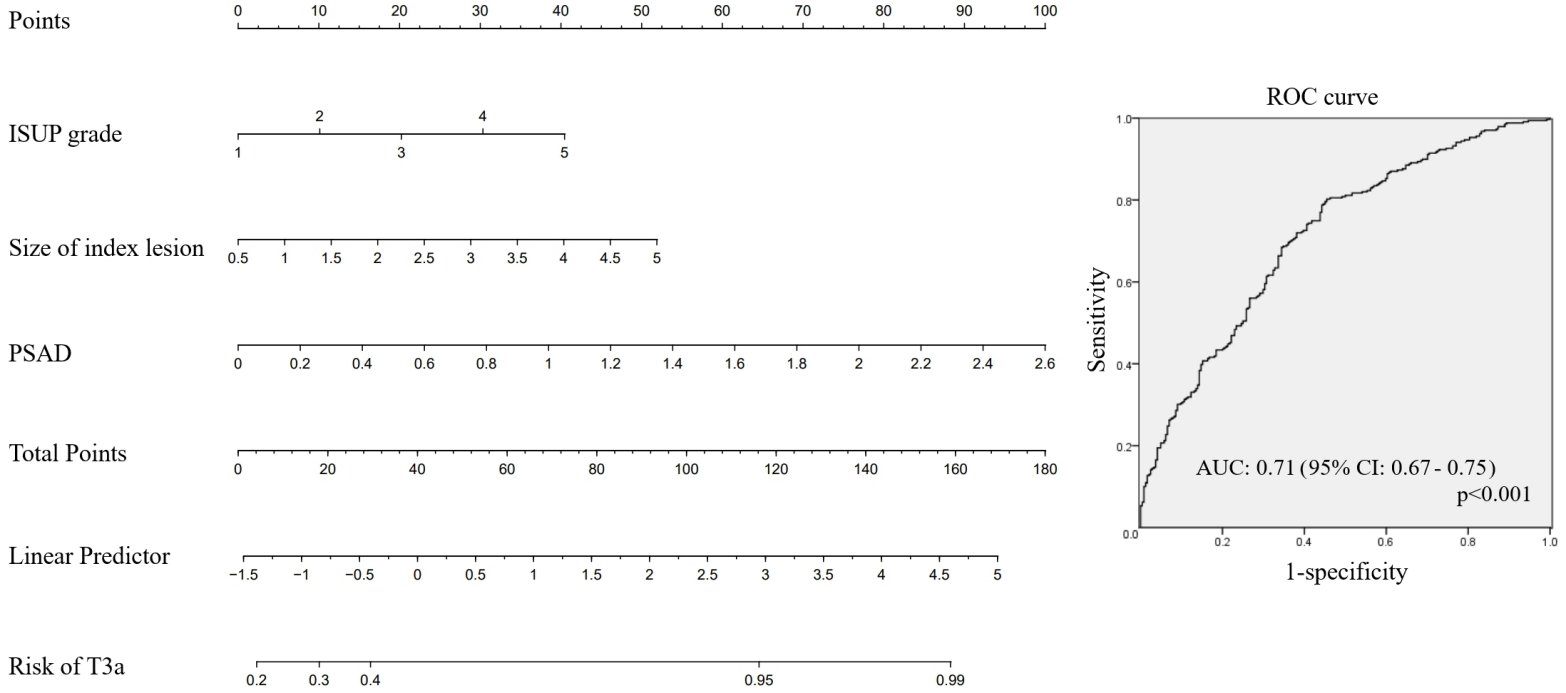
Nomogram for prediction of pT3a in patients with cT3a prostate cancer.

For prediction of pT3a among patients with cT3a PCa, a nomogram was developed using ISUP grade, index lesion size, and PSAD on prostate biopsy (**Figure 3**). The AUC of this nomogram was 0.71 (95% CI: 0.67–0.75, p<0.001).

## Discussion

Local staging is an essential step in determining the plan for PCa treatment (20). Clinical T3a PCa is classified as high risk because of the high recurrence rate, regardless of the PSA level or ISUP grade (4). Among the treatments for high-risk PCa, radical prostatectomy may provide a chance of cure, and its effectiveness as a part of multimodal treatment is well known (21). However, in prostatectomy of T3a PCa there is a high risk of a positive surgical margin and biochemical recurrence. Moreover, alterations in surgical strategy according to clinical stage can affect urinary continence and sexual potency (22). Satisfactory oncological and functional outcomes can be expected if the operation is performed with localization of radiologic ECE and a suitable surgical strategy; however, if the radiologically localized ECE region does not actually have capsular extension, the NVB may be unnecessarily sacrificed, which inevitably causes discomfort to the patient. Therefore, mpMRI findings play a very important role in determining the surgical treatment.

Recently, mpMRI for local staging using T2-weighted images and advanced techniques such as diffusion-weighted imaging and perfusion imaging has increasingly been employed. However, the accuracy of mpMRI for local staging is not all that high (23); in this study, 40.13% of patients with cT3a PCa were overestimated on mpMRI staging, 12.68% were under-estimated, and 47.19% were properly evaluated. Moreover, among the 2,757 RALP patients, 1,810 were diagnosed with cT2 PCa without neoadjuvant ADT. Among them 16.08%; pT3a (10.50%, 190/1810) or pT3b (3.76%, 68/1810), were under-estimated as localized prostate cancer; cT2a: pT3a (8.53%, 59/692) or pT3b (2.60%, 18/692); cT2b: pT3a (14.55%, 55/378) or pT3b (3.17%, 12/378); cT3c: pT3a (14.73%, 109/740) or pT3b (5.14%, 38/740). However, these data can be overestimated because we excluded patients with N1 in the pathological report.

The Partin table and the Memorial Sloan Kettering Cancer Center (MSKCC) nomogram are widely used tools for PCa risk classification (24, 25). The Partin table allows risk assessment for prostate cancer below cT2c, and the MSKCC provides survival and extent of disease probability using the history of androgen deprivation therapy and radiation therapy, age, PSA, Gleason score, and percentage of positive biopsy core. The MSKCC nomogram is a tool that can evaluate the probability of actual ECE when ECE is suspected on mpMRI. However, external validation of the prediction of ECE according to the MSKCC pre-prostatectomy nomogram reported AUCs of only 0.61–0.67 (26). In addition, the MSKCC nomogram includes the percentage of positive biopsy cores. Currently, a combined biopsy based on the pre-biopsy mpMRI is widely performed, with a multi-core targeted biopsy being performed on the index lesion. However, following the MSKCC pre-prostatectomy nomogram can result in the risk of ECE being overestimated in those patients who have undergone such a multi-core targeted biopsy.

In addition to clinical parameters such as PSA and Gleason score, mpMRI now plays a large role in clinical staging with the development of mpMRI technology and interpretation standards (27). The classification criteria include PIRADS v2.1 and a grading system with a Likert scale for evaluating ECE on mpMRI (16, 17). In this study, the uro-radiologists interpreted the mpMRI using PIRADS 2.1 and the Likert scale. However, interpretations according to these guides are subject to the subjective judgment of the radiologists involved. In addition, overestimation may occur if the index lesion is in contact with the capsule.

To supplement these aids, several nomograms for staging using mpMRI and clinical parameters have been reported. Bernard et al. assessed prediction of pT3-4 PCa using MSKCC, the Partin nomogram, and mpMRI, and reported that adding mpMRI was not significantly helpful for predicting locally advanced PCa (28). Gandaglia et al. reported that biopsy results and PIRADS ver. 2.0 were helpful for predicting adverse pathological features (29), although they evaluated adverse pathological features in patients with localized prostate cancer. However, Diamand et at. performed external validation of the nomogram of Gandaglia et al. and reported that the improvement in patient selection did not meet expectations (30). In addition, most of the studies to date have been on the evaluation of nodal status, ECE, and seminal vesicle invasion in only localized PCa.

Therefore, we conducted this study to find localized PCa in patients radiologically determined as T3a PCa. Currently, a large number of patients with locally advanced PCa choose RALP as a treatment. For RALP, differentiation between T2 and T3a is essential for determining the NVBS technique. In this study, factors affecting the classification of cT3a PCa into pathologic T2 T3a were evaluated, and a predictive model was developed. The significance of this study is that it might enable treatment with maximal preservation of functional outcomes while maintaining oncological outcomes in patients with cT3a PCa. The AUC of the nomogram in this study was 0.71 (95% CI: 0.67–0.75), which was far from optimal, but could be clinically helpful by suggesting a cut-off value. In this study, we retrospectively estimated that 13.16% (82/623) of the patients with cT3a PCa could have undergone NVB preservation. It is recommended to perform NVB preservation in such patients, but sufficient informed patient consent is important, and capsule violation must be very carefully avoided during surgery.

A limitation of this study is its retrospective design. Moreover, in the radiological evaluation, the degree of ECE on mpMRI, such as suspected, focal or extensive, could not be assessed in this study. However, the study enrolled a relatively large number of cT3a patients who received RALP. In addition, the nomogram has a relatively low AUC value and has not been externally validated. Despite these limitations, this study is the first to propose parameters for selecting patients with cT3a PCa (according to PIRADS ver. 2.1), who overestimated the preoperative radiological evaluation, could have undergone NVB preservation.

## Data availability statement

The raw data supporting the conclusions of this article will be made available by the authors, without undue reservation.

## Ethics statement

The studies involving humans were approved by Samsung Medical Center, Seoul, Korea. The studies were conducted in accordance with the local legislation and institutional requirements. The requirement for informed patient consent was waived by the board because of the retrospective nature of the study.

## Author contributions

WS: Data curation, Formal Analysis, Methodology, Project administration, Resources, Validation, Writing – original draft, Writing – review & editing. KK: Methodology, Resources, Writing – review & editing. JL: Methodology, Resources, Writing – review & editing. MK: Methodology, Resources, Writing – review & editing. HS: Methodology, Resources, Writing – review & editing. HJ: Methodology, Resources, Writing – review & editing. BJ: Methodology, Resources, Writing – review & editing. SS: Methodology, Resources, Writing – review & editing. SJ: Methodology, Resources, Writing – review & editing. HJ: Methodology, Resources, Writing – review & editing. JC: Conceptualization, Formal Analysis, Investigation, Methodology, Project administration, Software, Supervision, Visualization, Writing – original draft, Writing – review & editing.
